# Subgaleal Abscess Following Staphylococcal Cellulitis in a 10-Month-Old Child: A Diagnostic Challenge for the Clinician

**DOI:** 10.7759/cureus.12591

**Published:** 2021-01-09

**Authors:** Vijayakumary Thadchanamoorthy, Kavinda Dayasiri

**Affiliations:** 1 Clinical Sciences Department, Faculty of Health Care Sciences, Eastern University, Batticaloa, LKA; 2 Pediatrics, Mahaoya Base Hospital, Mahaoya, LKA

**Keywords:** cellulitis, subgaleal abscess, pustular lesion

## Abstract

Subgaleal abscess is an extremely rare and unusual presentation of head and neck infections and has been reported to occur following scalp infections, head trauma, sinusitis, septicemia, scalp monitoring, and surgical interventions. We report a 10-month-old child who presented with cellulitis around a pustule on the right forehead of three days duration. It was initially treated with oral cloxacillin for five-days. However, the child went on to develop a high continued fever on the day of discharge and remained febrile and unwell for seven days until a subgaleal abscess was identified and surgical drainage was performed. Pus cultures grew Staphylococcus aureus, which was sensitive to flucloxacillin. Following drainage of the abscess and change of antibiotics to intravenous flucloxacillin, fever completely subsided and the child made a complete clinical recovery. This report highlights the importance of having a high clinical suspicion of this rare complication in children with continuing high spikes of temperature following skin infections in the head region.

## Introduction

A subgaleal abscess is rare in healthy children and is reported as a potentially dangerous complication of untreated or partially treated infections in the scalp. It commonly occurs following infection of a subgaleal hematoma, which could be secondary to a skull fracture, incidental minor injuries to the scalp, birth trauma, and either hair pulling or vigorous hair combing [1-7]. In the newborn, it has been reported following needle aspiration of a subgaleal hematoma [8]. Herein, the authors have reported a child who had a subgaleal abscess secondary to impetigo of the forehead.

## Case presentation

A 10-month-old, previously healthy child presented with cellulitis around a pustule on the right side of the forehead for a three-day duration. It was very painful, disturbing his sleep; in addition, cellulitis was extending over the same duration. The rash was not associated with fever and the child’s activity, and feeding had been satisfactory. As it was an obvious localized lesion (2.5 cm x 3 cm), oral cloxacillin was commenced for impetigo-associated cellulitis of the forehead, and investigations were not performed. On the following day, after having normal observations and good clinical response, he was planned for discharge. However, on the same day, he developed high-grade fever with chills and rigors and became irritable and unwell.

Subsequently, the diagnosis was reviewed and the child was reassessed. The physical examination was normal apart from tenderness over the back of the scalp. The investigation revealed that high white blood cells (16x103/ cumm) with neutrophil leukocytosis (80% of white blood cells), elevated C-reactive protein (CRP; 12 mg/dL), and erythrocyte sedimentation rate (40mm/first hour). Ultrasound of the scalp and brain was normal. Chest X-ray was normal. Since the diagnosis was not apparent, the child was commenced on broad-spectrum intravenous antibiotics as for sepsis. Despite being on intravenous antibiotics for three days, his fever continued and increased up to 39.2 °C. Subsequently, the antibiotic was changed to intravenous cefotaxime as increasing white blood cells (18.3x103/cumm, N-76%) and CRP (24 mg/dl) were noticed. Cerebrospinal fluid (CSF) analysis and blood culture were negative. Although fever spikes started to decline following the change in antibiotics, the fever did not reach the baseline. The mother reported pain mainly over the right side of the head, as he was crying while handling his head. Tenderness was elicited over the same region. A repeated ultrasound scan of the scalp showed a subgaleal abscess on the right side of the nape (5 cm x 5 cm). The abscess was drained under ultrasound guidance, and the pus that was sent for culture grew Staphylococcal aureus. Based on antibiotic sensitivity, the antibiotic was changed to intravenous flucloxacillin and continued for five days. Following drainage of the abscess and change of antibiotics to intravenous flucloxacillin, fever completely subsided, and the child made a complete clinical recovery.

## Discussion

The subgaleal space is a potential space between the skull periosteum and the scalp and formed by the epicranial aponeurosis, which extends from the supraorbital ridge to the zygomatic arches and auricular muscles laterally and to the cervical triangle muscles posteriorly. Increased laxity of the loose connective tissues of the subaponeurotic space in children has been thought to be allowing for greater mobility of the scalp relative to the cranium. Emissary veins connecting dural sinuses and superficial scalp veins reside in this space along with loose connective tissue. Infections can potentially seed through both hematogenous spread and direct bacterial invasion leading to abscess formation [9-10].

A subgaleal abscess has been reported to be caused by multiple pathogens. A neonatal subgaleal abscess usually follows invasive fetal monitoring with the placement of scalp electrodes and the most common pathogen is Staphylococcus epidermidis [11]. Staphylococcus aureus has been reported in a subgaleal abscess that follows scalp infections, similar to this child [12]. Other reported pathogens include Escherichia coli [13], methicillin-resistant Staphylococcus [14], Streptococcus pyogenes [15], Eikenella corrodens [16], Salmonella enteritidis [17], and Burkholderia pseudomallei [18].

Computed tomography (CT) brain identifies hypodense collections and allows better visualization for the aspiration of subgaleal fluids. Operative debridement can be performed alternatively for extensive subgaleal abscesses [9-10]. The subgaleal abscess in this child was drained under ultrasound guidance and further surgical interventions were not required.

In the reported child, infection of the subgaleal space could be secondary to direct extension rather than hematogenous spread since the child had been indicating scalp pain throughout the period, and later, the infection likely localized to form a subgaleal abscess. Identification of the subgaleal abscess was backed by the atypical course of the swelling. This atypical presentation was supported by persistently increased inflammatory markers and continuing high spikes of temperatures without any other focus of infection.

## Conclusions

The authors have reported on a child who developed a subgaleal abscess caused by Staphylococcus aureus following impetigo and cellulitis of the forehead. The mechanism of subgaleal abscess infection in this child is likely to be direct spread via the scalp. It is important to have a high clinical suspicion of this rare complication in children with continuing high spikes of temperature following skin infections in the head region.
